# Exploring the hidden world of RNA viruses with a transformer-based tool

**DOI:** 10.1016/j.patter.2024.101095

**Published:** 2024-11-08

**Authors:** So Nakagawa, Shoichi Sakaguchi

**Affiliations:** 1 Department of Molecular Life Science, Tokai University School of Medicine, Isehara, Japan; 2 Division of Omics Sciences, Institute of Medical Sciences, Tokai University, Isehara, Japan; 3 Division of Interdisciplinary Merging of Health Research, Micro/Nano Technology Center, Tokai University, Hiratsuka, Japan; 4 Department of Microbiology and Infection Control, Faculty of Medicine, Osaka Medical and Pharmaceutical University, Takatsuki, Japan

## Abstract

Hou and He et al.^1^ developed a new RNA virus identification tool named LucaProt, a transformer-based bioinformatics software using sequence and structural characteristics of RNA-dependent RNA polymerases (RdRPs), which are essential for almost all RNA viruses. LucaProt can identify RdRPs from highly diverse RNA viruses, unveiling the hidden RNA virosphere.

## Teaser

Hou and He et al. developed a new RNA virus identification tool named LucaProt, a transformer-based bioinformatics software using sequence and structural characteristics of RNA-dependent RNA polymerases (RdRPs), which are essential for almost all RNA viruses. LucaProt can identify RdRPs from highly diverse RNA viruses, unveiling the hidden RNA virosphere.

## Main text

Many RNA viruses have been identified by analyzing metatranscriptomes, datasets created by sequencing the total RNA content of an entire environmental or organismic sample without isolation of individual species. However, due to the high genomic diversity of RNA viruses, it remains difficult to comprehensively identify RNA viruses from metatranscriptomes through bioinformatics analyses. Since most RNA viruses are believed to share a self-replicating enzyme known as RNA-dependent RNA polymerase (RdRP), which has a critical role in RNA viral replication, the identification of RdRP sequences has been a focal point in discovering RNA viruses. Nevertheless, even though RdRPs perform an essential function, their amino acid sequences are highly diverse, including within their functionally important domains. This presents a challenge for sequence-based identification of RNA viruses in metatranscriptome data, particularly for those RNA viruses that differ from well-known RNA viruses.^2^

In a recent paper in *Cell*, Hou and He et al. present a transformer-based bioinformatics tool for RdRP identification called LucaProt^1^ (https://github.com/alibaba/LucaProt), a Python-based software supporting both CPU and GPU (CUDA) configurations licensed under the Apache License (Version 2.0). LucaProt has integrated two channels to process amino acid sequence compositions and protein structure information using a transformer-based deep learning approach to examine whether a given amino acid sequence contains viral RdRP. 5,979 well-characterized RdRPs and 229,434 non-RdRP protein sequences were used as positive and negative samples, respectively. Using the same dataset, the authors also created a BLAST and hidden-Markov-model-based RdRP search program named ClstrSearch (https://github.com/alibaba/LucaProt/tree/master/ClstrSearch). By comparing LucaProt with ClstrSearch and other major RdRP identification programs, they found that LucaProt achieved better recall, precision, and false-positive rates of RdRP identification, enabling a more comprehensive and large-scale exploration of RNA viruses. The results suggest that transformer-based models could effectively handle long-range relationships across amino acid sequence sites of RdRPs, leading to better prediction performance than other methods. This work is further evidence of the growing popularity of transformer models, which have been shown to surpass the performance of recurrent neural network and convolution neural network models in various applications.^3^^,^^4^

With metatranscriptomic sequencing data of 10,487 samples, LucaProt revealed 161,979 potential RNA viruses belonging to 180 RNA viral supergroups, which are comparable to existing viral classes and phyla as defined by the International Committee on Taxonomy of Viruses (https://ictv.global/). Among the identified RNA viruses, there is one Nido-like virus showing a 47.3-kb-long genome, one of the longest RNA viruses identified to date. The newly discovered RdRPs were verified through three different approaches. First, the key RdRP motifs were confirmed to be present in the alignment. Second, several RdRP sequences found in this study were amplified by real-time PCR, while DNA was simultaneously confirmed to be absent, suggesting that these exist only as RNA isoforms. Finally, the predicted RdRP protein structures of the representative supergroups were similar to those of known RdRPs rather than those of other self-polymerases, such as reverse transcriptase and eukaryotic DNA polymerase. These findings suggest that LucaProt can accurately identify RdRPs from various metatranscriptomic data, even when they exhibit low sequence similarity to known RNA virus RdRPs. Interestingly, the newly identified RNA viruses discovered were predominantly found in environmental samples, and “marker viral species” that are abundant in each specific environment could also be detected.

Ultimately, LucaProt could assist in exploring the identification of RNA viruses from various metatranscriptome data. We have personally evaluated seven RdRP identification tools and databases, including LucaProt,^1^ using double-stranded RNA sequencing data.^5^ LucaProt identified the largest number of potential RdRP sequences, 375 candidates, significantly surpassing the 254 and 247 RdRP sequences found by the second and third highest-performing tools, Palmscan^6^ and NeoRdRp2,^5^ respectively, although these RdRP candidates have not been examined in depth in our study.

While numerous RdRP-containing sequences have been identified in recent years, there are several limitations to RdRP studies for RNA virus identification. For example, some RNA viruses exhibit a segmented genome structure, where certain contigs contain RdRP while others do not. As Hou and He et al. mentioned, identifying RNA viral contigs lacking RdRPs may likely be inherently impossible with current methods. Moreover, several studies showed the presence of RNA viruses or virus-like sequences lacking RdRP, such as Delta and Epsilon viruses^7^ and viroid-like Obelisks,^8^ which cannot be identified through RdRP-based searches. Nonetheless, despite the current limitations, we believe LucaProt has a significant impact on this research field and is expected to facilitate the discovery of various new RNA viruses, expanding our knowledge of the diversity and evolution of RNA viruses. Altogether, the next important steps in this research field, we believe, involve further investigation into the biological and ecological roles of these newly discovered RNA viruses.

## Acknowledgments

The authors thank the participants of the first RdRp Summit^2^ for their valuable discussions.

## Declaration of interests

The authors declare no competing interests.

## Declaration of generative AI and AI-assisted technologies in the writing process

During the preparation of this work, the authors used Grammarly and ChatGPT in order to improve language and readability. After using this tool/service, the authors reviewed and edited the content as needed and take full responsibility for the content of the publication.
